# Supplementary material for: Self-correction in science: The effect of retraction on the frequency of citations
Source: PLoS One. 2022 Dec 7;17(12):e0277814. doi: 10.1371/journal.pone.0277814 (PMC9728909; doi:10.1371/journal.pone.0277814)
Supplement: Attachment — Submitted filename: PLOS1_response to reviewers_rev1.docx [file pone.0277814.s002.docx]

Response to Reviewers

Title: Self-correction in science: The effect of retraction on the frequency of citations

by Anton Kühberger, Daniel Streit, & Thomas Scherndl

This letter contains our reaction to the comments of the reviewers. We are very grateful for their insightful comments which made the manuscript much better.

Reviewer #1.

#1: no action necessary

#2: no action necessary

#3: no action necessary

#4: no action necessary

#5: Review Comments.

The reviewer suggested the following points to round up the paper:

a) Causality is established only by temporality, but not by specificity or consistency. The reviewer opted for establishing a few more measures of association and suggested a subgroup analysis of the retracted papers (retracted within <=2 years vs < 2 years; pp. 12-13). The revised version of the paper entails such a subgroup analysis. We found an interesting pattern of results, depicted in the new Figure 5, and discussed on pp. 12-13. The analysis offers some interesting new insights and strengthens the causality assumption.

b) The reviewer argues that we have not explained why an increase in the control group annual citations (figure 4) should be expected. On p. 14 we do this now. In addition, we explain why there is a bump specifically after the year of retraction.

c) The reviewer argues that we should report some additional data on the papers. We do this on p. 7. Frequencies of papers and publication years are visible in Fig. 2.

d) We corrected minor grammatical errors.

Reviewer #2.

#1: no action necessary

#2: no action necessary

#3: no action necessary

#4: no action necessary

#5: Review Comments

a) The reviewer asked for providing actionable suggestions/ recommendations for ensuring that retracted articles are not further highlighted via citations. Specifically, (s)he opted for defining the role technology to combat bad practices. We included a new section detailing what and how we think the problem could be alleviated. Broadening the reviewers comment, we include also a discussion of why not only the citation of retracted papers after retraction, but citation even before retraction is a problem, when later retraction is not yet known. We also broaden the discussion by relating the discussion to citations of nonreplicating research. The Discussion was extended by more than two pages and contains some 15 new references.

b) The reviewer suggests analysis scientific domains. We cannot do this since we did not systematically collect data on domains.

c) The reviewer asks for provision of appropriate and adequate references to hierarchical linear mixed-effects models, and why we used it here. We do this on p. 9.

d) The reviewer recommends being more explicit on inherent biases in retracted publications, especially with respect to current topics of interest. We do this in the revision, in the extended discussion section (see pp. 15-17).

e) The reviewer asks what can be done to ensure that high quality standards are encouraged so that the percentage of retracted papers reduce with time? We are more explicit on this in our new discussion section (pp. 12 – 16).

f) The reviewer sees strong need to capture the retracted papers during the review process and bring it forward to the journal editor's attention before accepting the same for publication. Again, we are more specific on this in the Discussion.

In sum, we responded to the comments of the reviewers and we added some additional data and analysis. We hope the revision will be successful in making the paper publishable.

Yours sincerely

Anton Kühberger
